# Effect of the Aspect Ratio and Tilt Angle on the Free Convection Heat Transfer Coefficient Inside Al_2_O_3_–Water-Filled Square Cuboid Enclosures

**DOI:** 10.3390/nano12030500

**Published:** 2022-01-31

**Authors:** Redhwan Almuzaiqer, Mohamed ElSayed Ali, Khaled Al-Salem

**Affiliations:** Mechanical Engineering Department, College of Engineering, King Saud University, Riyadh 11421, Saudi Arabia; ralmuzaiqer@ksu.edu.sa (R.A.); kalsalem@ksu.edu.sa (K.A.-S.)

**Keywords:** convection heat transfer, nanofluids in enclosures, experimental heat transfer

## Abstract

This experimental study provides a comprehensive investigation of natural convection heat transfer inside shallow square cuboid enclosures filled with aluminum oxide–water nanofluid at four different volume concentrations: 0.0%, 0.2%, 0.4%, and 0.8%. Two square cuboid enclosures were used with sizes 30 × 30 × H cm^3^, where H is the inside thickness of the enclosures. This led to two different enclosure aspect ratios (κ = H/30 = 0.033 and 0.066). Four inclination angles to the horizontal position of the enclosures were used: 0°, 30°, 60°, and 90°. The crucial thermophysical properties of the synthetic nanofluid were obtained. The thermal conductivity of the nanofluid was measured experimentally at various volume concentrations. Furthermore, the viscosity and density were also measured experimentally at temperatures ranging from 15 to 40 °C as a function of the volume concentration. The heat transfer data were generated by heating the lower surface of the enclosure using a uniform flexible heat flux heater. The opposite surface was cooled using an air fan. The results of the experimental physical parameter measurements show that the percent of maximum deviation in thermal conductivity with those in the literature were 6.61% at a 1.0% volume concentration. The deviation of dynamic viscosity was between 0.21% and 16.36% at 0.1% and 1% volume concentrations, respectively, and for density it was 0.29% at 40 °C and a 1% volume concentration. The results showed up to a 27% enhancement in the Nusselt number at an angle of 60° and a 0.4% volume concentration in the largest aspect ratio (κ = 0.066). However, for the low aspect ratio enclosure (κ = 0.033), there was no noticeable improvement in heat transfer at any combination of volume concentration and inclination angle. The results show that the inclination angle is a significant factor in natural convection only for large aspect ratio enclosures. Furthermore, for large aspect ratio, the Nusselt number increased until the angle approached 60°, then it decreased again.

## 1. Introduction

Natural convection in enclosures occurs in a wide range of industrial applications and engineering systems: solar collectors, thermal insulation of buildings, and cooling systems for nuclear reactors and electronic devices [1,2,3,4,5,6,7]. Because natural convection is less efficient than forced convection, it should be further investigated to be improved. Through many kinds of industrial thermal processes, it is necessary to add, remove, or exchange heat. Therefore, enhancing the rate of heating and cooling inside an industrial operation will help save energy, decrease the processing time, and increase the performance life of machinery. One strategy for enhancing heat transfer that has received tremendous attention from studies over the past decade is the use of nanofluids [8,9,10,11]. The term “nanofluid” refers to a suspension of conductive nanoparticles in a base fluid such as water. A nanofluid has considerably better thermal conductivity than a base fluid. According to the most recent studies in the field, nanofluids may also increase heat transfer in cavities and channels. Despite the number of studies undertaken, the mechanism by which a nanofluid could enhance natural convection in a cavity is still not completely understood. Certain conclusions of the research are contradictory for several reasons including a lack of valid experimental data, fundamental theoretical investigations, and precise numerical simulations. To simplify simulations, several researchers have assumed a homogenous mixture for nanofluid flow, which is a two-phase flow with a significant relative drift or slip velocity between particles and the base fluid [12,13]. In addition, it is possible that the appropriate thermophysical property correlations are not employed in certain cases.

A comprehensive review of studies on free convection in a cavity was carried out by Pandey et al. [14]. The shape effect of the internal cavity, such as a square, circular, and elliptical cylinder, on free convection heat transfer was summarized. Free convection heat transfer inside two water-filled square enclosures was investigated experimentally by Ali et al. [15]. Two different aspect ratios, κ (length/height) = 7.143 and 12.0, were used. The Nusselt numbers was correlated with the modified Rayleigh numbers for both enclosures in the range $4\times 10^{6}<Ra_{H}^{*}<3.5\times 10^{8}$. They observed that the Nusselt number increased with an increase in the modified Rayleigh number for each of the two enclosures with a higher Nu at a small aspect ratio (κ = 7.143). Almuzaiqer et al. [16] investigated the effect of tilt angle on free convection inside an enclosure filled with water. The Nusselt number reached a maximum at 60° at a fixed modified Rayleigh number for all four tilt angles considered: 0°, 30°, 60°, and 90°. The Nusselt number was found to be higher at any tilt angle other than at a zero tilt angle with an enhancement range of 7.92–62.38%, depending on the modified Rayleigh numbers and the tilt angle. The same trend was observed through other numerical studies [17,18,19] that showed that the Nusselt number reached its maximum at a certain tilt angle and then decreased again. Ma et al. [20] used numerical simulations and parameter sensitivity analyses to investigate the performance of fluid flow and heat transfer in rectangular microchannels including the key physical properties of the fluids and the different parameters of the microchannels. They found that at low Reynolds number conditions, the number of channels and the Reynolds number have a significant impact on heat transfer. However, when the Reynolds number increases, the number of channels is the key factor influencing the heat transfer and flow in microchannel heat sinks. Zhao et al. [21] presented a comprehensive overview of graphene-based studies of energy conversion, energy storage, and heat transfer. A nanofluid of graphene nanoparticles can also be effectively used in heat exchangers and other heat transfer devices. In their review, they reported that when hybrid graphene nanoplatelets and silver in a water base fluid were used in the rectangular duct, the maximum Nusselt number enhancement was 32.7% and the friction factor increased by 1.08 times at 0.1% concentration (by mass) and a Reynolds number of 17,500. Hu et al. [22] investigated experimentally and numerically the natural convection heat transfer in a vertical square enclosure filled with an alumina nanofluid. Their study showed an enhancement of 2% in the Nusselt number at a low nanoparticle concentration of a 1% mass fraction. However, at a 2% concentration, they found no enhancement and a degradation occurred at a 3% concentration. Ali et al. [23,24] investigated natural convection heat transfer in vertical circular cavities using Al_2_O_3_–water nanofluid at different volume concentrations for heating either from the top or the bottom of the cavity. While heating from the top, alumina–water nanofluid had a lower Nusselt number than the base fluid. On the other hand, when heating from the bottom, the heat transfer coefficients increased with an increase in the volume concentration up to a maximum point; then, they decreased as the volume concentration increased further. The heat transfer coefficient increased by a maximum of 40% for the shallow enclosure at κ (height/diameter) = 0.0635 and only by 8% for κ = 0.127. Solomon et al. [25] studied the effect of cavity aspect ratio on free convection in alumina–water nanofluid-filled rectangular cavities. The aspect ratio of the cavity has an impact on both the heat transfer coefficient and the Nusselt number. A total of seven volume concentrations (0.0%, 0.1%, 0.2%, 0.3%, 0.4%, 0.5%, and 0.6%) were used at a set of temperatures (∆T = 20, 30, 40, and 50 degrees Celsius) between cold and hot surfaces. At low concentrations, nanofluids demonstrated a slight increase in Nu over that of the base fluids, up to 5%, whereas at high volume concentrations, a decrease in Nu was observed. Choudhar and Subudhi [26] investigated turbulent free convection in an Al_2_O_3_–water-filled cavity with different aspect ratios of 0.3–2 and 5 for Rayleigh numbers in the range of 10^7^ < Ra < 10^12^ for very low volume concentrations of 0.01% and 0.1%. It was observed that Nu was enhanced by 29.5% for lower particle concentrations, 0.01 vol.%, where deterioration was caused by increasing the viscosity and decreasing the Brownian motion. The effect of inclination angles on free convection in an enclosure filled with Cu–water was numerically analyzed by Abu-Nada and Oztop [27]. An enhancement was observed in the Nusselt number of approximately 33% at a 90° tilt angle with a Rayleigh number of 1000 and for a 0.1% nanofluid concentration. Heris et al. [28] studied the free convection in a cube with a side length of 100 mm. The effect of the tilt angle on free convection was observed. Their study used 0°, 45°, and 90° tilt angles and various types of nanofluids of Al_2_O_3_, TiO_2_, and CuO with turbine oil as a base fluid. However, the influence of the inclination angle on the aspect ratio was not examined. They concluded that no enhancement was observed when using different nanoparticles in turbine oil as a base fluid. In other words, the Nusselt numbers of turbine oil as a base fluid were higher than other nanofluids using turbine oil as a base fluid. The natural convection of double-walled carbon nanotubes–water nanofluid in a cuboid cavity was experimentally and numerically studied in [29] at a set of different temperatures. It was observed that the heat transfer coefficients and Nusselt numbers reached a maximum at a 0.05% concentration and then decreased as the volume concentration increased. The natural convection heat transfer of SiO_2_–water nanofluid in a rectangular cavity was studied experimentally by Torki and Etesami [30] at various concentrations and inclination angles. It was found that using SiO_2_–water nanofluid at low concentrations (0.1%) did not significantly improve natural convection heat transfer coefficients; however, the coefficient of natural convection was reduced at volume concentrations of more than 0.5%. Heat transfer rates also decreased with inclination angle, and Nusselt numbers have a maximum value at a 0° tilt angle. The free convection heat transfer in enclosures with CuO–water nanofluid that was heated from the right side and cooled from the top was numerically analyzed by Bouhalleb and Abbassi [31], where five small aspect ratios were investigated (i.e., 0.08, 0.1, 0.125, 0.25, and 0.5). The effect of Rayleigh number, aspect ratio, and inclination angle on flow patterns and energy transport was investigated. They found an improvement in heat transfer when using CuO–water nanofluid. The Nusselt number reached its maximum at volume concentrations of 2% and 2.5% for aspect ratios of 0.5 and 0.25 and 0.125, 0.1, and 0.08, respectively. It was also observed that Nu reach its maximum at 30°, then decreasing as the angle increased.

As seen in the literature survey presented above, experiments on the natural convection heat transfer of nanofluids in enclosures that investigate the effect of tilt angle and aspect ratio are limited. Most of the studies in the literature involve only 2D numerical analyses; however, the present study employed 3D analyses using wide enclosures, and the thermophysical properties were determined experimentally and compared to those in the literature. The current experimental investigation aimed to determine the influence of the inclination angle and the aspect ratio on free convection heat transfer using an aluminum oxide–water nanofluid in square cuboid cavities at two different aspect ratios. This extensive study will be valuable for future theoretical, numerical, and practical studies in the field of natural convection inside cavities.

## 2. Material and Methods

### 2.1. Nanofluid Formulation

**Figure 1:**
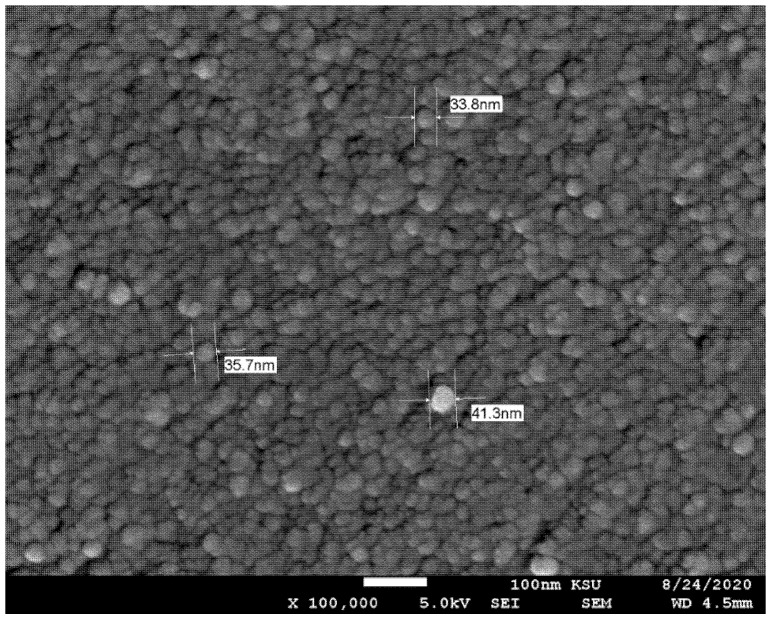
SEM images of nanoparticles illustrate aggregation to larger sizes.

Dispersed γ-Al_2_O_3_ (20% by weight) in water was obtained from Nanostructure and Amorphous Material Inc. in Los Alamos, NM, USA. Table 1 lists the specifications provided by the manufacturer. The required volume concentration of dispersed nanofluid was achieved by diluting it with distilled water. Four different volume concentrations of Al_2_O_3_–water nanofluid were prepared: 0.0%, 0.2%, 0.4%, and 0.8%. Another three additional volume concentrations (i.e., 0.1%, 0.5%, and 1%) were prepared for the purpose of evaluating the thermophysical properties of the nanofluid in order to provide a clear trend of the measured values. A magnetic stirrer (230 V, 50–60 HZ, 17 × 17 cm panel, 500 watt) was used for two hours in newly diluted nanofluid to prevent nanoparticle agglomeration. Additionally, the aggregation of the nanoparticles was disrupted using an ultrasonic agitation probe with high power output (Qsonica Q-700; 40 kHz, 700 watts, 3 s on and 1 s off, Newtown, CT, USA) for eight hours [32]. The stability of nanofluids is frequently investigated using a scanning electron microscope (SEM) [33,34]. As seen in Figure 1, using a JEOL JSM-6360 A SEM [Tokyo, Japan], the Al_2_O_3_ nanoparticles can be seen to be spherical in shape and exhibit a slight aggregation, where the size of the particles was approximately 10 nm.

### 2.2. Thermophysical Properties of the Nanofluids

To characterize the prepared Al_2_O_3_–water nanofluid, the thermophysical properties, including thermal conductivity, dynamic viscosity, and density, were measured experimentally at different volume concentrations. The thermophysical properties of nanofluids also depend on the operating temperature of the nanofluids. Therefore, the temperature should be recorded during the measurement of nanofluid properties. The thermophysical properties of nanofluids are prerequisites for determining the coefficient of heat transfer, Nusselt number, and Rayleigh number [35]. Experimental measurements were obtained for the thermal conductivity, dynamic viscosity, and density of the nanofluids. On the other hand, correlation equations available in the literature can be utilized to compute other thermophysical properties, such as the specific heat and thermal expansion coefficient, due to the low solid volume fraction in the utilized mixture [36]. The thermophysical properties of the base fluid (water) and the nanoparticles (Al_2_O_3_) are listed in Table 2. Table 3 lists the specifications of the used base fluid, which was provided by a twice distilled water machine (HAMILTON, WSC/4, Kent, United Kingdom) available at the College of Engineering, Mechanical Engineering Lab. The lab instruments used in this study are shown also in Table 4.

#### 2.2.1. Thermal Conductivity

The transient hot-wire (THW) approach was used to evaluate the thermal conductivity of the nanofluid. It is a reliable and rapid method [4,10,29,35,36,38]. In this case, the KD2 Pro thermal property analyzer (Decagon Devices, Inc., Pullman, WA, USA) was used. A 60 mm long and 1.27 mm thick stainless-steel KS-1 thermal conductivity sensor was suitable for measuring the thermal conductivity of the liquid, and it was placed in a vial of nanofluid and connected to a power supply. It should be mentioned that before starting the measurements, the thermal conductivity analyzer equipment was calibrated using distilled water, and the maximum error was found to be approximately 5%. To assure the accuracy of the obtained data, each measurement was repeated ten times, and the average was taken. All measurements were carried out at an ambient temperature of 23 °C.

#### 2.2.2. Dynamic Viscosity and Density

A kinematic viscometer from Anton Paar (SVM 2001, Graz, Austria), which has a high accuracy of ±5% and a wide range of application, was used to measure the dynamic viscosity and density. The kinematic viscometer device was first calibrated with distilled water. Furthermore, in order to ensure the reliability of the results, the tests were repeated three times at temperatures ranging from 15 to 40 °C as well as at different volume concentrations: 0.1%, 0.2%, 0.4%, 0.5%, 0.8%, and 1%.

#### 2.2.3. Specific Heat and Thermal Expansion

The following two equations were used to calculate the constant specific heat and thermal expansion coefficient, [4,29,35,36]:

(1) $${\left(\rho C_{p}\right)}_{eff}=\left(1-\varphi \right){\left(\rho C_{p}\right)}_{bf}+\varphi {\left(\rho C_{p}\right)}_{p}$$

(2) $$\beta_{nf}=\varphi \beta_{P}+\left(1-\varphi \right)\beta_{bf}$$

### 2.3. Experimental Setup

**Figure 2:**
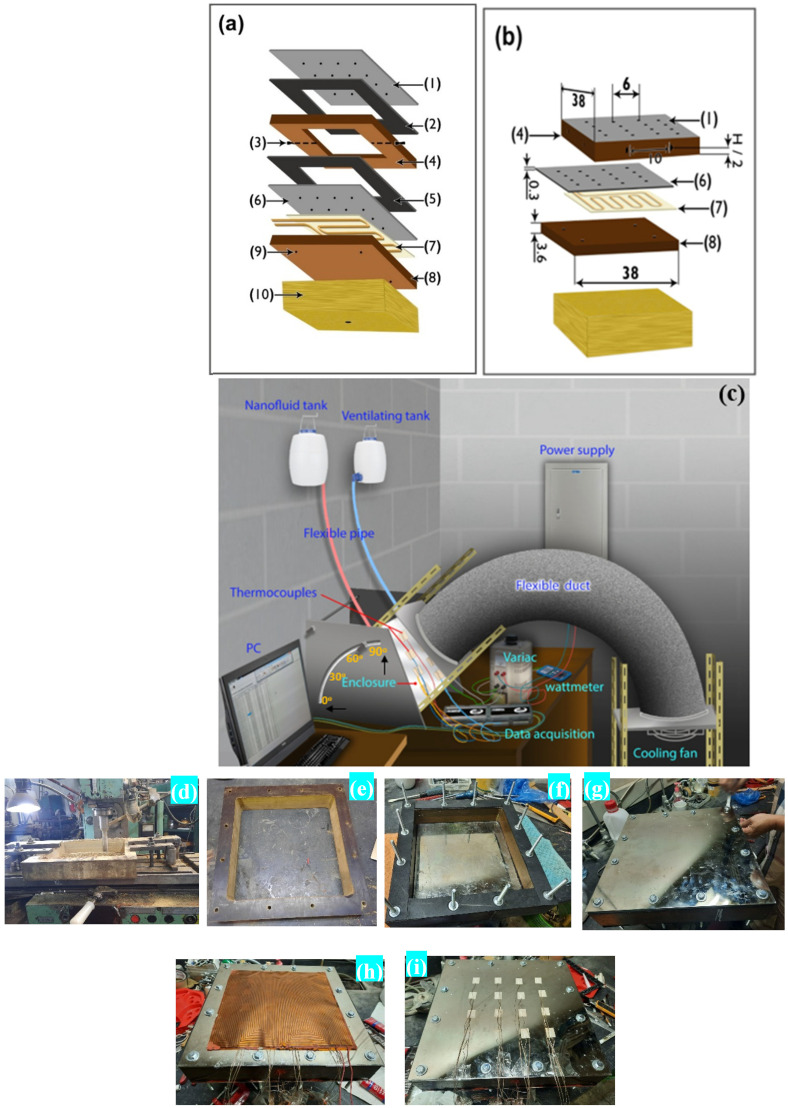
Experimental test rig for handling the enclosure: (**a**,**b**) enclosure parts—(**1**) upper copper plate (cold surface), (**2**) sheet of a gasket, (**3**) two-directional valve, (**4**) square cuboid enclosure, (**5**) sheet of the gasket, (**6**) bottom copper plate (hot surface), (**7**) heater, (**8**) Bakelite insulator sheet, (**9**) thermocouples, and (**10**) insulation cover; (**c**) complete assembled setup; (**d**–**i**) images taken during preparation of the enclosures.

The experimental test rig was designed to test the natural convection heat transfer for two square cuboid enclosures filled with aluminum oxide–water nanofluid. Figure 2a–c provide a detailed illustration of the enclosures, while Table 5 lists the component’s materials and the dimensions of each enclosure. Figure 2d–i show images taken during the preparation of the enclosures. The enclosure frame (4) and part (8) were. made of Bakelite (k = 0.15 W/(m ∙ K) [39]. The outside dimensions of all enclosures were 38 × 38 cm^2^. Two sheets of gaskets (2 and 5) were placed between the copper plates and the enclosure to prevent any potential leakage issues. It should be noticed that the copper plates (1 and 6) were coated with a layer of nickel at a thickness of 0.3 mm in order to prevent corrosion, which may occur in the future. Part (4) of the enclosure was equipped with two valves (3), one for filling the nanofluid and the other for air ventilation as seen in Figure 2a,c. At the bottom of the copper plate (6) (the hot surface), a flexible foil heater (7) of 30 × 30 cm^2^ with a maximum thickness of 2.54 × 10^−4^ m was installed. A 3 cm thick Bakelite plate (8) was used to insulate the other side of the heater. There were 16 thermocouples (Type-K) (shown as dots in Figure 2a,b) put on the upper and lower copper plates (1 and 6) to measure their surface temperatures. On the lower Bakelite surface (8), four additional thermocouples (9) were attached. Eight additional thermocouples were placed around each enclosure’s sidewalls, two on each side and one on the outer surface, and the other inserted through the side and leveled at the inner surface to monitor any heat loss through the enclosure’s side. The thermocouple signals were transferred to a computer using a data acquisition system for thermal analysis. A voltage regulator was used to control the electrical power provided to the heater and generate heat transfer data. A wattmeter was used to determine the consumed power at each run.

### 2.4. Experimental Procedure

**Figure 3:**
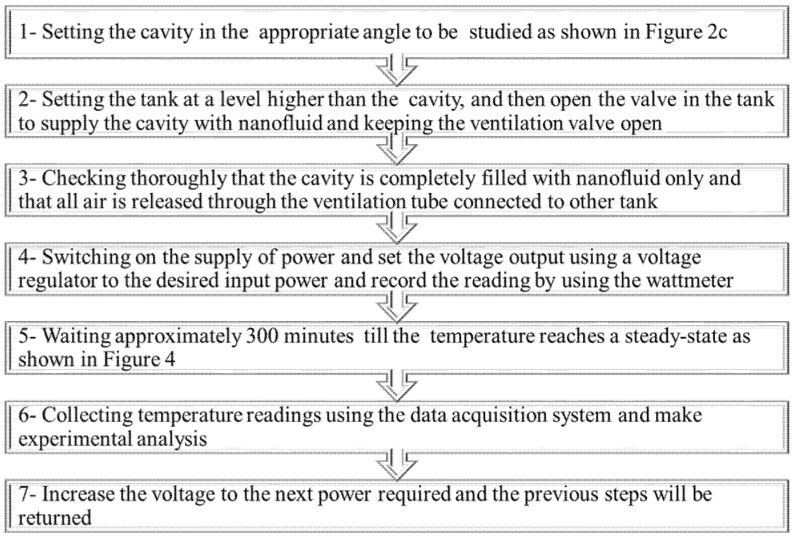
Experimental steps.

**Figure 4:**
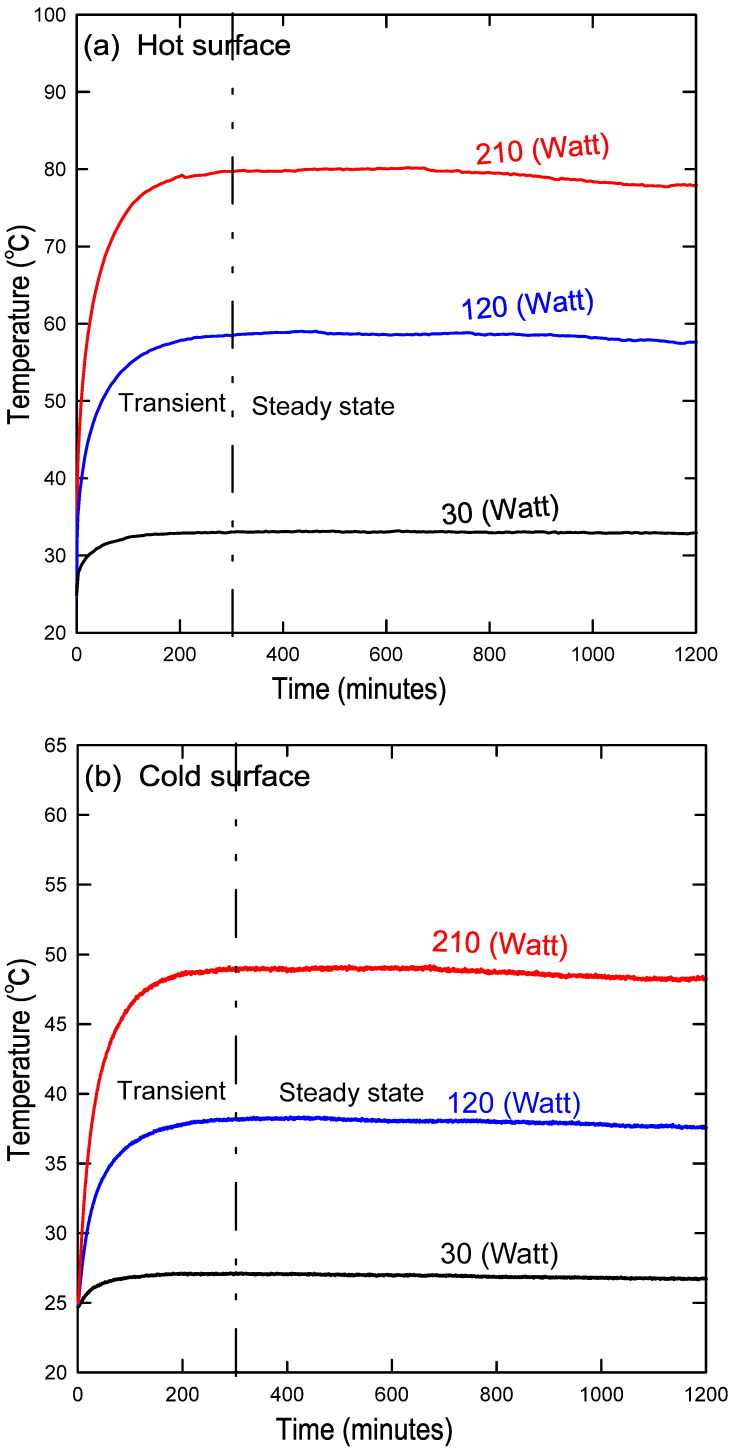
Steady-state condition reached after almost 300 min at the (**a**) hot copper surface and the (**b**) cold copper surface.

Figure 3 summarizes the preliminary procedures that must be performed before starting the experiment and collecting data. It is important also to ensure that there are no gases or bubbles present while filling the cavity with nanofluid. This is accomplished by shaking the cavity repeatedly until all possible bubbles remaining inside the cavity have escaped through the ventilation tank. Furthermore, after each experiment with a specified nanofluid concentration, the cavity was washed three times with water to ensure that no residue from the previous concentration was left over. Figure 4a, b show the steady-state temperature for a variety of heat fluxes at both the hot and cold surfaces, respectively. This figure indicates that the system reached a steady state at approximately 300 min.

### 2.5. Experimental Analysis

**Figure 5:**
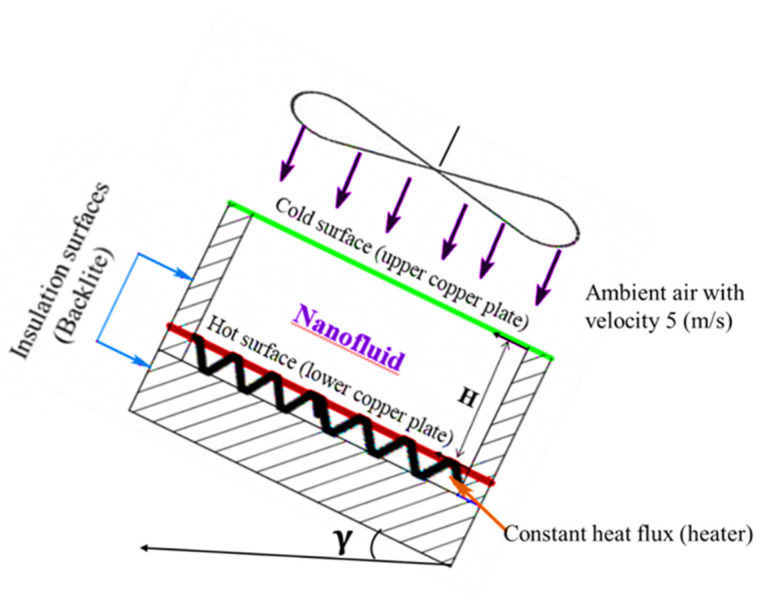
Schematic of the experimental setup with boundary conditions.

Heat transfers occurred through the constant heat flux heater by conduction via the lower copper plate, natural convection through the nanofluid inside the cavity, conduction through the top copper plate, and by forced convection through ambient air. In addition, the amount of heat that may be lost through the sides and the bottom of the Bakelite plate were calculated. The heat lost by conduction through the Bakelite plate below the heater and from the Bakelite sides was obtained as 3.1% and 7.9% at most, respectively. Figure 5 shows a schematic of the experimental setup with boundary conditions, where the lower copper surface was subject to a constant heat flux, the side walls were insulated, and the upper copper surface was subject to ambient air at 5.0 m/s. The heat transfer by radiation was ignored since the maximum temperature of the nanofluid did not exceed 80 °C and the working fluid was water [40]. The amount of heat transfer can be calculated from Equations (3)–(5).

(3) $$Q_{total}=IV=Q_{Bkp}+Q_{Bks}+Q_{ET}$$

(4) $$Q_{Bkp}=A_{Bkp}k_{Bkp}\frac{{\bar{T}}_{avg\cdot h}-{\bar{T}}_{Bkp}}{\delta_{Bkp}}$$

(5) $$Q_{Bks}=A_{Bks}k_{Bks}\frac{T_{inside}-{\bar{T}}_{Bks}}{\delta_{Bks}}$$

where *Q_total_*, $Q_{Bkp}$, $Q_{Bks}$, and $Q_{ET}$ are the total input electrical power, the rate of heat lost through the insulated lower and side surfaces by conduction, and the rate of heat transfer through the enclosure, respectively. The surface areas $A_{Bkp}$ and $A_{Bks}$ stand for the insulation surfaces covering the heater and the side walls, respectively.

#### 2.5.1. Average Heat Transfer Coefficient $\left(h_{\mathrm{avg}}\right)$

At steady-state condition, the total rate of heat transfers through the lower heated surface up to the outer cold surface was calculated using:

(6) $$Q_{ET}=\frac{∆\bar{T}}{\sum R}=\frac{{\bar{T}}_{avg\cdot h}-{\bar{T}}_{avg\cdot c}}{\sum R}$$

It should be noted that Equation (6) uses the average surface temperatures of both the hot and cold copper surfaces, respectively. The cavity thermal resistance was calculated using:

(7) $$\sum R={\mathrm{lower}\;R}_{\mathrm{Copper}}+R_{\mathrm{fluid}}+{\mathrm{upper}\;R}_{\mathrm{Copper}}$$

where

(8) $$R_{\mathrm{Copper}}=\frac{∆x_{\mathrm{Copper}}}{A_{\mathrm{Copper}}{\;k}_{\mathrm{Copper}}},\;R_{\mathrm{fluid}}=\frac{1}{A\;h_{\mathrm{avg}}}$$

and the copper surface area ($A_{\mathrm{Copper}})$ is equal to the natural convection area (A) of the cavity, and $k_{\mathrm{Copper}}$ = 394 W/m ∙ K [39]:

(9) $$Q_{ET}=\frac{{\bar{T}}_{avg\cdot h}-{\bar{T}}_{avg\cdot c}}{2\frac{∆x_{\mathrm{Copper}}}{A_{\mathrm{Copper}}{\;k}_{\mathrm{Copper}}}+\frac{1}{A\;h_{\mathrm{avg}}}}$$

Using Equation (9), the average heat transfer coefficient through the cavity can be calculated as:

(10) $$\;h_{\mathrm{avg}}=\frac{1}{\left[\left(\frac{{\bar{T}}_{avg\cdot h}-{\bar{T}}_{avg\cdot c}}{Q_{ET}}-2\frac{∆x_{\mathrm{Copper}}}{A_{\mathrm{Copper}}{\;k}_{\mathrm{Copper}}}\right)A\right]}$$

Furthermore, the average Nusselt and the modified Rayleigh numbers were [41]:

(11) $${\bar{Nu}}_{H}=\frac{\;h_{\mathrm{avg}}\cdot H}{k}$$

(12) $$Ra_{H}^{*}{}^{\;}=\frac{g\beta Q_{ET}H^{4}}{k\upsilon \alpha A}$$

The thickness (H) of the cavity was used as a characteristic length in Equations (11) and (12).

#### 2.5.2. Uncertainty Calculations

Estimating the experimental uncertainty was performed using the engineering equation solver (EES) [42]. It was necessary to repeat some of the experiments more than once to check the overall trend of the data. The uncertainty of the surface area and temperature were calculated as 0.001 m^2^ and 0.1 °C, respectively. The wattmeter’s handbook was used to determine the wattmeter’s voltage and current measurement accuracy. The readings of the temperature were recorded using a data acquisition system. An average of 30 temperature scans was calculated at each specified heat flux. The EES also provides the capability to propagate the uncertainty of experimental data to provide uncertainty estimates of calculated variables. The method used by EES for determining the uncertainty follows Reference [43]. Table 6 summarizes the uncertainty calculated for different quantities.

## 3. Results and Discussion

Experimental measurements of several of the thermophysical properties of the used nanofluids are presented and compared with published correlations in the literature. Then, the results of heat transfer through the enclosures are discussed.

### 3.1. Thermophysical Properties Analysis

#### 3.1.1. Thermal Conductivity Analysis

**Figure 6:**
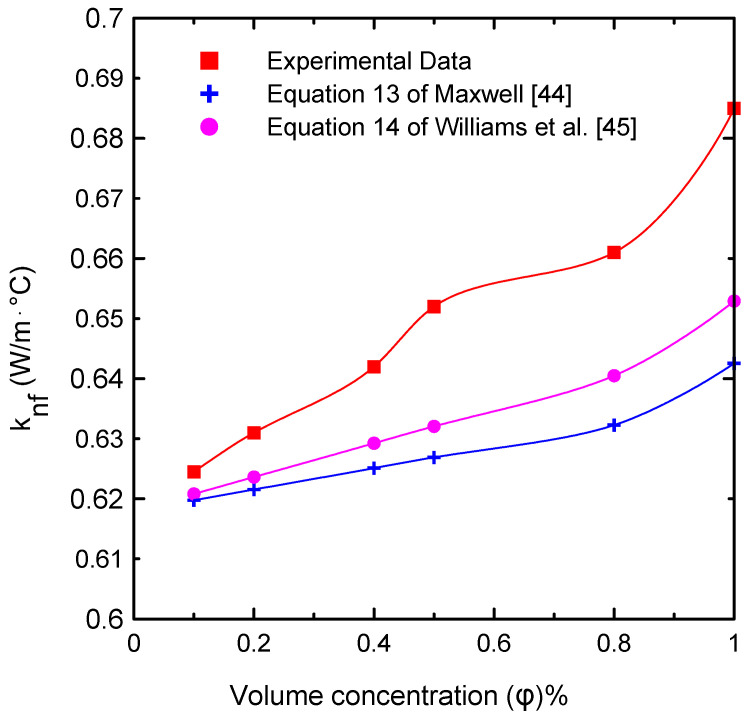
Comparison of the thermal conductivity of the nanofluid experimental data with the predicted correlations [44,45] available in the literature at different volume concentrations.

**Figure 7:**
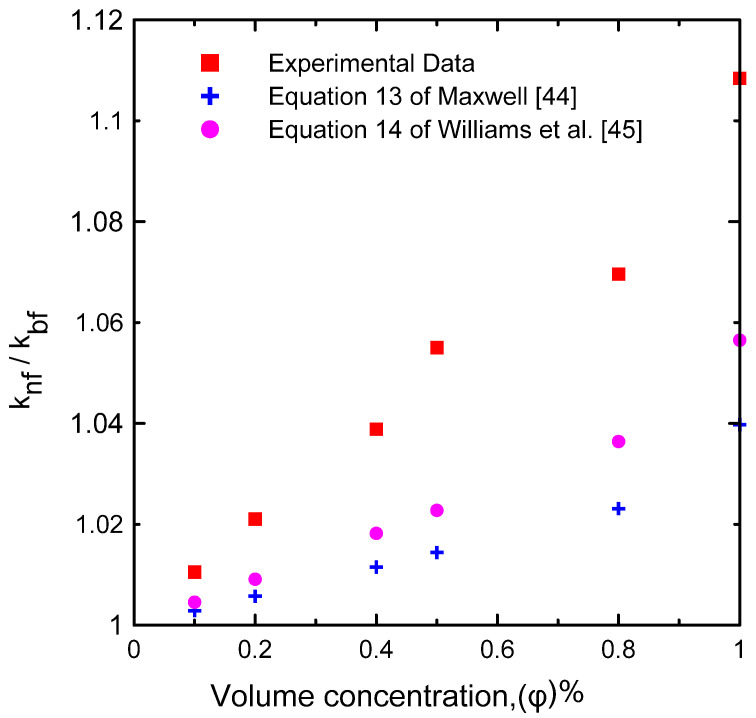
The effective thermal conductivity of the experimental results compared to the correlations available [44,45] in the literature at various volume concentrations.

Figure 6 illustrates the effects of the nanofluid volume concentrations (φ = 0.0, 0.1, 0.2, 0.4, 0.5, 0.8, and 1 Vol.%) on thermal conductivity at ambient temperature (23 °C). The results were compared with published correlations in the literature (Equation (13) of Maxwell [44] and Equation (14) of Williams [45]). This figure shows that the difference between the measured data and the correlations was approximately 6.61% at most, because thermal conductivity is affected by many factors such as the shape and size of the used nanoparticles in preparing the nanofluid. The effective thermal conductivity (*k_nf_/k_bf_*) with respect to volume concentration is shown in Figure 7. There were no substantial changes in effective thermal conductivity at low concentrations of solid particles. On the contrary, for high solid volume fractions, the effective thermal conductivity increased significantly. This can be attributed to the increasing number of collisions, as the number of solid particles increased in the base fluid in addition to the Browning motion [9,22,32,33]. Table 7 shows the deviation between the current experimentally measured thermal conductivity and those of Equation (13) of Maxwell [44] and Equation (14) of Williams [45] at different percentages of nanoparticle volume concentrations at ambient temperature. The maximum deviation obtained was 6.61% at a 1% volume concentration, which imparts confidence in the current experimental measurements.

(13) $$k_{eff}=\frac{k_{p}+2k_{bf}+2\varphi \left(k_{p}-k_{bf}\right)}{k_{p}+2k_{bf}-\varphi \left(k_{p}-k_{bf}\right)}\;k_{bf}$$

(14) $$k_{eff}=k_{bf}\;\left(1+4.5503\varphi \right)$$

#### 3.1.2. Dynamic Viscosity

**Figure 8:**
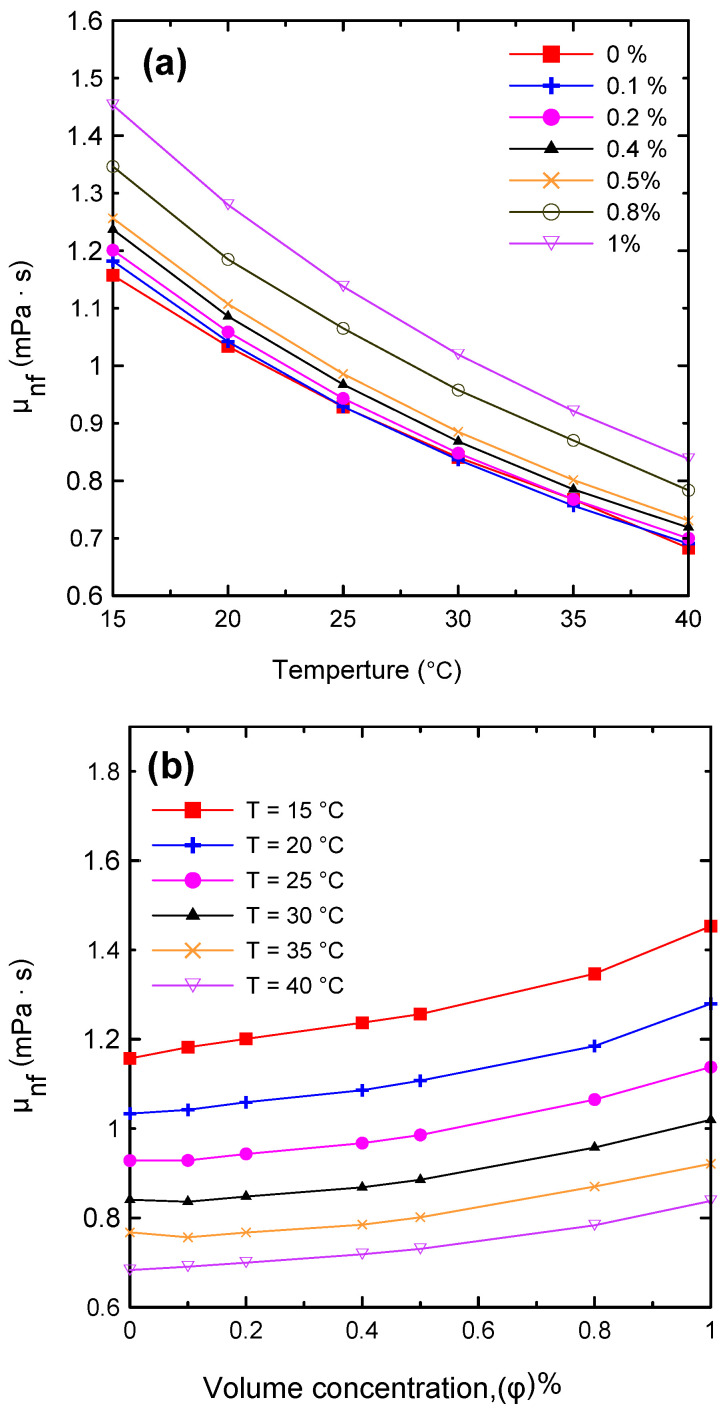
Variations in the nanofluid’s dynamic viscosity with (**a**) temperature and (**b**) volume concentration.

**Figure 9:**
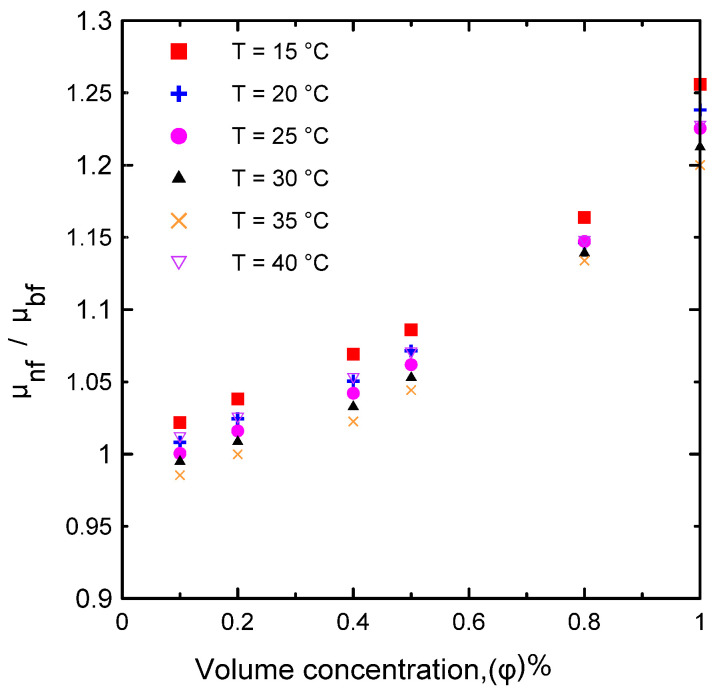
Relative dynamic viscosity of the current experimental data for different volume concentrations and at different temperatures.

**Figure 10:**
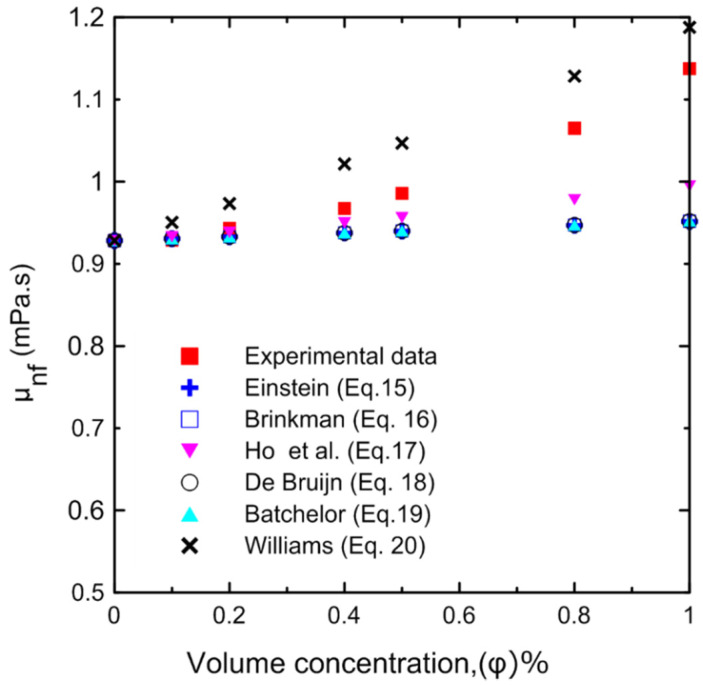
Experimentally measured nanofluid viscosity compared to different correlations in the literature at different volume concentrations at 25 °C.

The dynamic viscosity of the Al_2_O_3_–water nanofluid was examined in relation to two primary factors, namely, the temperature of the nanofluid and the volume concentration. The dynamic viscosity was evaluated at seven different volume concentrations (Ф = 0.0, 0.1, 0.2, 0.4, 0.5, 0.8, and 1 vol.%) and at a temperature range of 15–40 °C. It was observed that the nanofluid’s dynamic viscosity decreased as the temperature increased, as seen in Figure 8a, because as the temperature of the fluid increased, the intermolecular forces decreased [10,29]. Furthermore, as the concentration of the nanofluid increased, the viscosity increased, as shown in Figure 8b, due to the increased friction between the fluid and the nanoparticles. The relative value of dynamic viscosity (*μ_nf_/μ_bf_*) variation with respect to nanoparticle volume concentrations as a function of temperature is depicted in Figure 9. Table 8 lists a variety of theoretical and empirical models for predicting the viscosity of nanofluids. Figure 10 illustrates the results of those models as a function of volume concentration at 25 °C compared to the current experimental results. It is quite clear that as the concentration of nanoparticles increased, the deviation from the experimental points diverged. The range in deviation was between 0.21% and 16.36%, which corresponded to 0.1% and 1% volume concentrations, as shown in Table 9, at 25 °C. Consequently, it is important to determine the thermophysical parameters experimentally to ensure that the correlation utilized is as close as possible to the experimental results, otherwise using an incorrect correlation can have a significant impact on heat transfer coefficient estimates [10,29,46].

#### 3.1.3. Density

**Figure 11:**
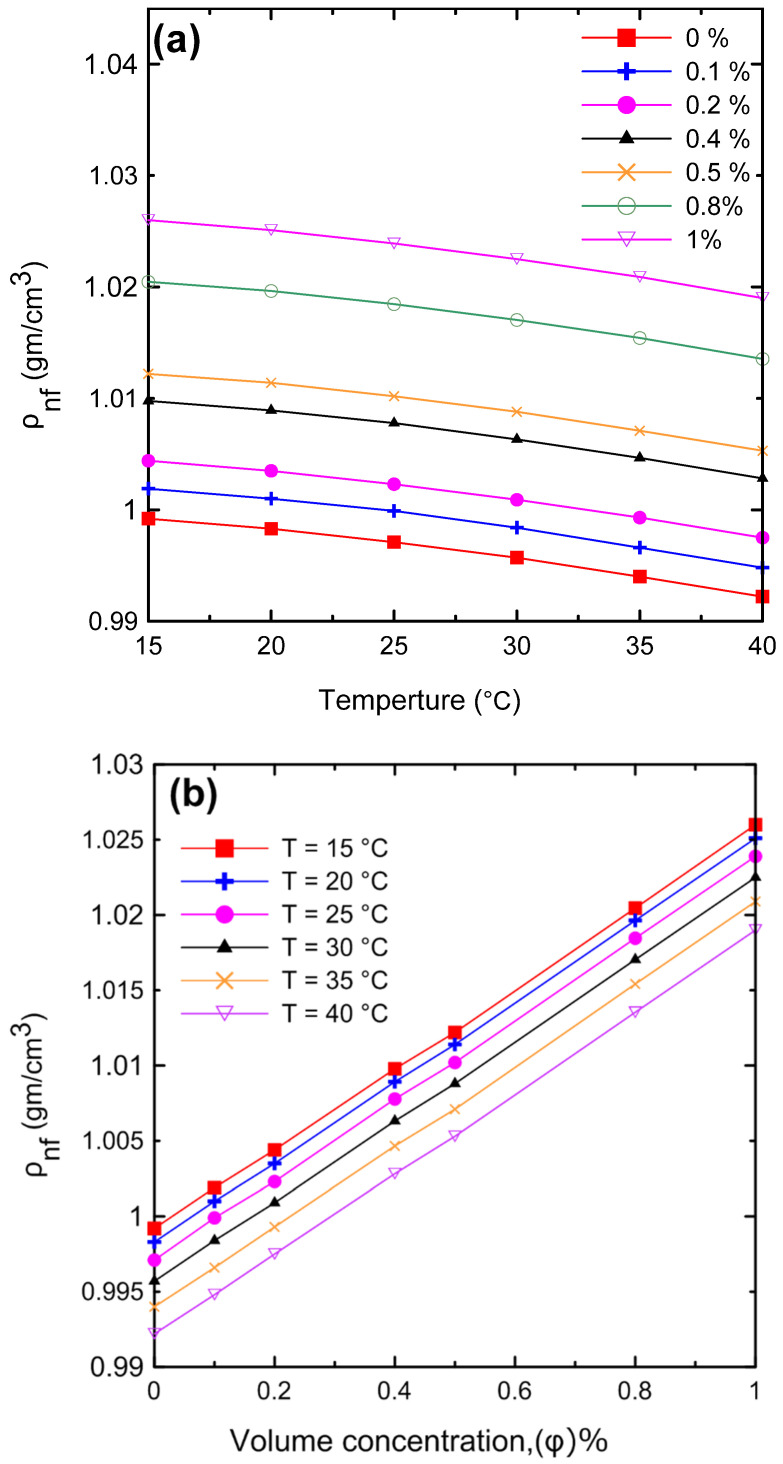
The density of the nanofluids for the experimental results as a function of (**a**) temperature and (**b**) volume concentration.

**Figure 12:**
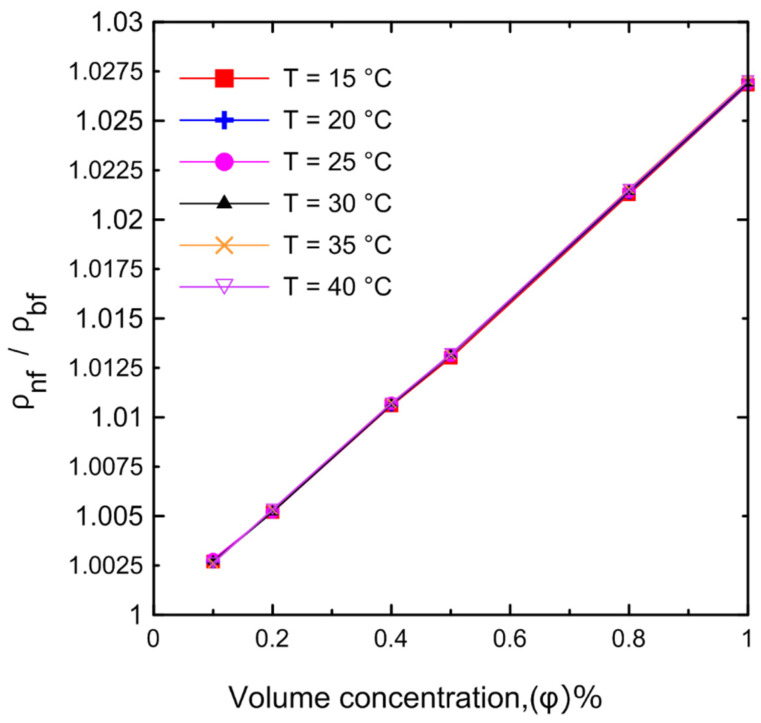
Relative nanofluid density for the experimental results at different volume concentrations as a function of temperature.

**Figure 13:**
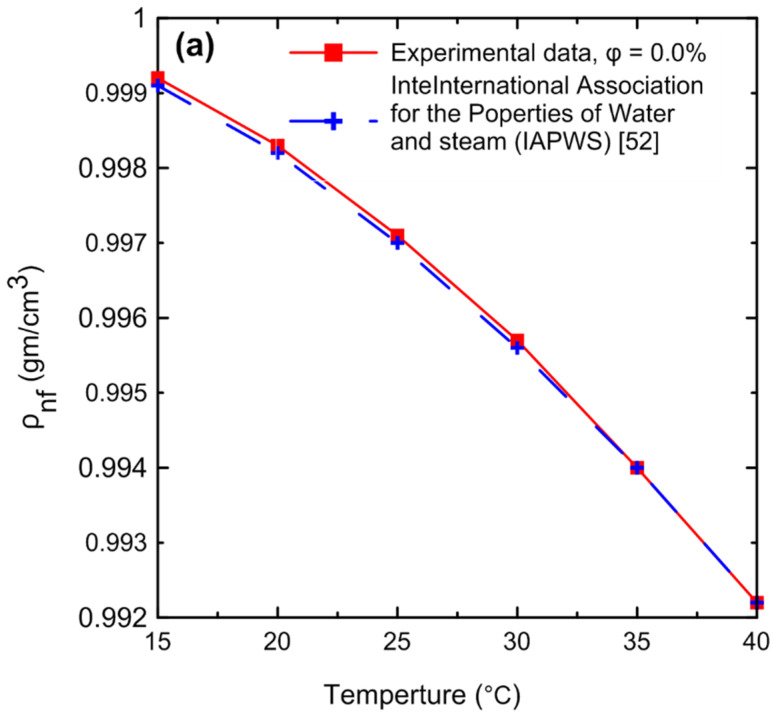

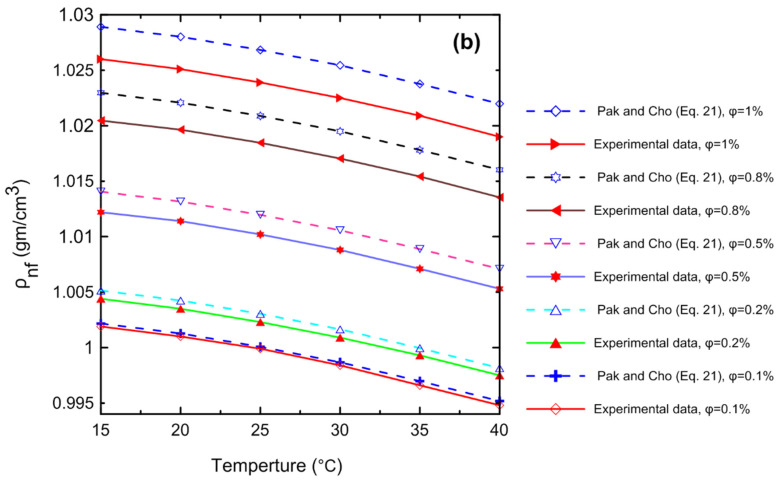
Comparison of the density experimental results for (**a**) pure water with (IAPWS) [52] and (**b**) nanofluid at different concentrations using the theoretical density Equation (21) of Pak and Cho [53].

A comprehensive study of density measurements was conducted for the Al_2_O_3_–water nanofluids with particle volume concentrations of Ф = 0.0, 0.1, 0.2, 0.4, 0.5, 0.8, and 1 Vol.%. The experimental results for the density of the nanofluid as a function of (**a**) temperature and (**b**) volume concentration are shown in Figure 11. The density of the nanofluid decreased as the temperature increased as shown in Figure 11a. At lower temperatures, in general, the liquid molecules lose energy, slowing down and resulting in closer liquid molecules and a decrease in liquid volume. As the temperature of the liquid increases, it expands or grows in volume, so that the temperature of the liquid increases and its density decreases. The density of the nanofluid increased as the volume concentration increased as shown in Figure 11b. Because more nanoparticles were added to the base fluid, the mass will increase; thus, the density of the nanofluid will also increase. Figure 12 demonstrates the relationship between the relative nanofluid density and the volume concentration. It can be clearly seen that increasing the volume concentration led to a greater relative density and that the volume concentration had a greater impact on the relative density than the temperature. In order to verify the instrument’s accuracy and reliability, it was calibrated by measuring the density of pure water at various temperatures and comparing the results to those available in the literature as shown in Figure 13a. An excellent agreement was observed between the measurement data and the data published by the International Association for the Properties of Water and Steam (IAPWS) [52]. In addition, the experimental results of the nanofluid at different temperatures were compared with theoretical density Equation (21) of Pak and Cho [53], with a maximum difference of 0.3% at 1% (Vol.) as shown in Figure 13b.

(21) $$\rho_{nf}=\left(1-\varphi \right)\;\rho_{bf}+\varphi \;\rho_{p}$$

Table 10 shows a comparison of the experimentally measured density and Equation (21) of Pak and Cho [53] for Al_2_O_3_–water nanofluid at different temperatures and concentrations.

### 3.2. Heat Transfer Analysis

**Figure 14:**
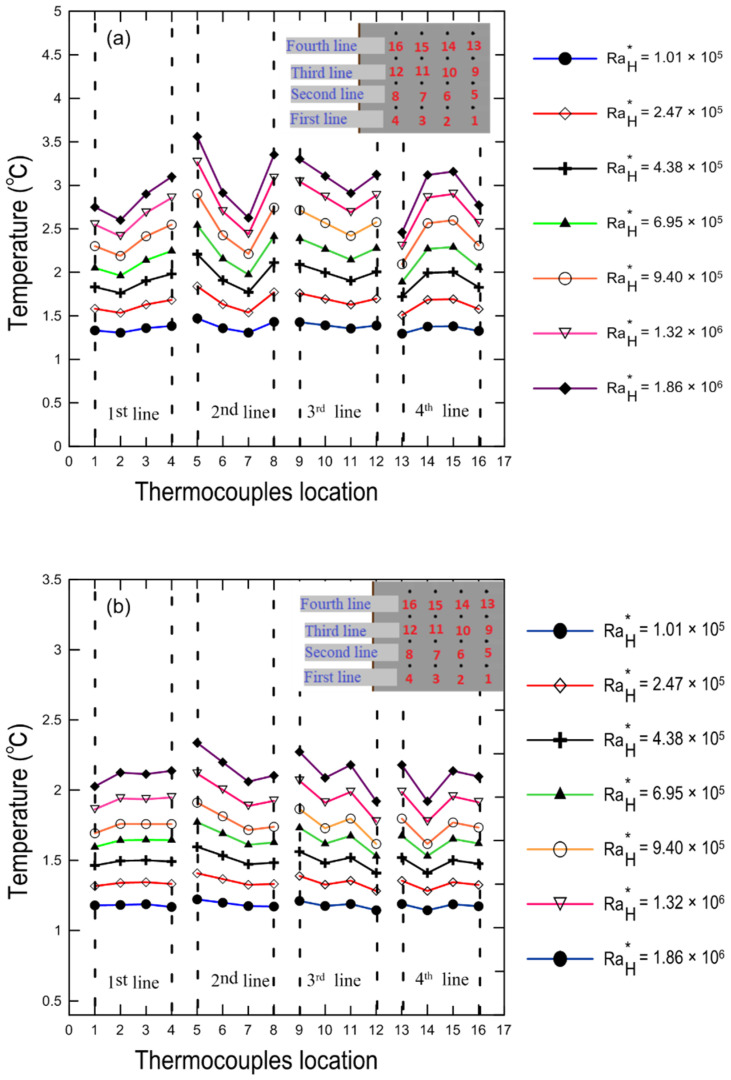
Temperature profiles along the thermocouple location on the copper surfaces at a 0° tilt angle for κ = 0.033: (**a**) the hot bottom surface; (**b**) the cold top surface.

Temperature profiles normalized by the ambient temperature for the hot and cold surfaces of the enclosure (k = 0.033) are shown in Figure 14a,b, respectively, for a variety of modified Rayleigh numbers at a 0.8% volume concentration and 30 degree tilt angle. It is clear that temperature increased as the modified Rayleigh number increased.

It should be noted that since we used an attached flexible heater with uniform heat flux at the bottom of the lower stainless-steel plate, we did not expect to have a uniform surface temperature. The reason for this could be attributed to the fact that the copper coated plates are not massive and highly conductive material (k = 394 W/m ∙ K), [41]. There may be no exact thermal contact between the heater and the hot surface at some spots, which leads to the existence of contact resistance between the heater and the copper plate.

**Figure 15:**
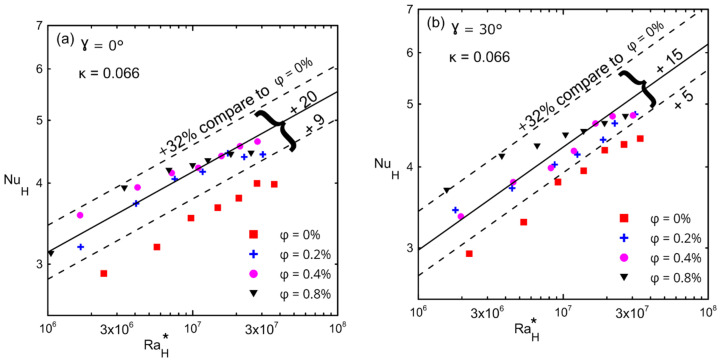

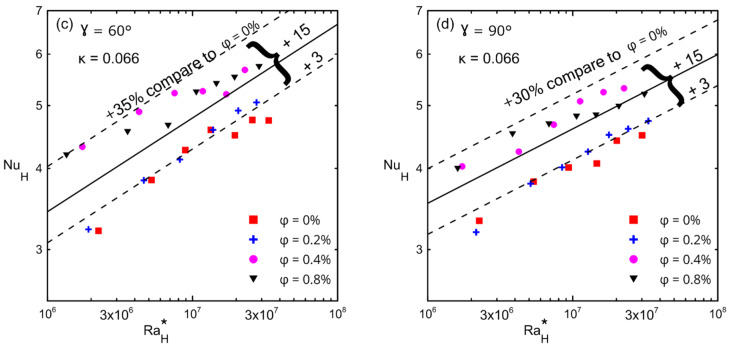
Nusselt number versus modified Rayleigh number for Al_2_O_3_–water nanofluid for enclosure number 1 (κ = 0.066) showing the effect of volume concentrations: (**a**) 0°; (**b**) 30°; (**c**) 60°; (**d**) 90°.

The average Nusselt number with respect to the modified Rayleigh number is shown in Figure 15, (a) γ = 0°, (b) γ = 30°, (c) γ = 60°, and (d) γ = 90° for different inclination angles and for different volume concentrations (ϕ) for the large enclosure (#1, κ = 0.066). The lower and upper dashed lines in these figures present the minimum and maximum enhancements in the Nusselt number over that of a zero concentration (symbol ■). The solid lines in these figures present the average enhancement due to the fact that all concentrations (greater than 0%) were between the minimum and maximum enhancements. Table 11 presents the minimum, maximum, and average enhancement of Nusselt numbers corresponding to different inclination angles for all concentrations. Furthermore, Table 11 shows the percentage of enhancement in the Nusselt number corresponding to each concentration with respect to a zero concentration at different angles. These figures also show that as the modified Rayleigh number increased, the convection velocity increased too, which led to more kinetic energy that allowed the Brownian motion to be more effective [23,24,25,29,30]. This figure indicates that the average enhancement reached a maximum at 0° (20%) and then reached a uniform enhancement of 15% at the other tilt angles for all concentrations at all range of the modified Rayleigh numbers.

**Figure 16:**
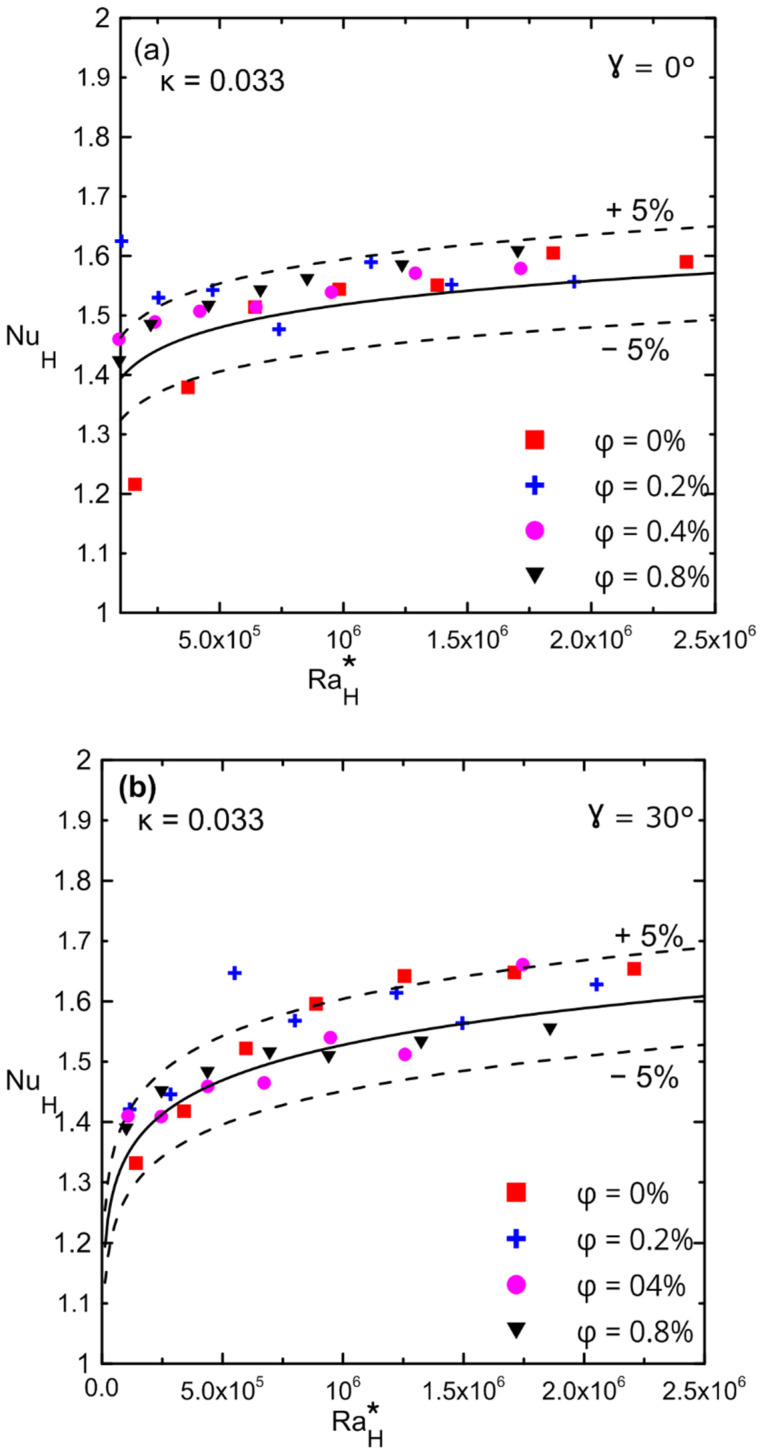
Nusselt number versus modified Rayleigh number for Al_2_O_3_–water nanofluid for enclosure number 2 (κ = 0.033) showing the effect of volume concentration: (**a**) 0°; (**b**) 30°.

Figure 16a,b show the variation in the Nusselt numbers versus the modified Rayleigh numbers for different volume concentrations for enclosure number 2 (κ = 0.033). The Nusselt number dd not change significantly for zero and thirty degrees of an inclination angle for all ranges of the modified Rayleigh numbers. The presence of nanofluid had a weak effect on the Nusselt number due to the low aspect ratio enclosure, which reduced convection and allowed only pure conduction to take place in the system (Nu is of the order one). Furthermore, since a small volume concentration was used, the percentage of enhancement in the thermal conductivity of nanofluid was not significant enough to have a significant impact even on pure conduction. Comparison between Figure 15a,b and Figure 16a,b, confirms that the enhancement in Nu was due to the effect of decreasing the aspect ratio for the same applied heat fluxes.

**Figure 17:**
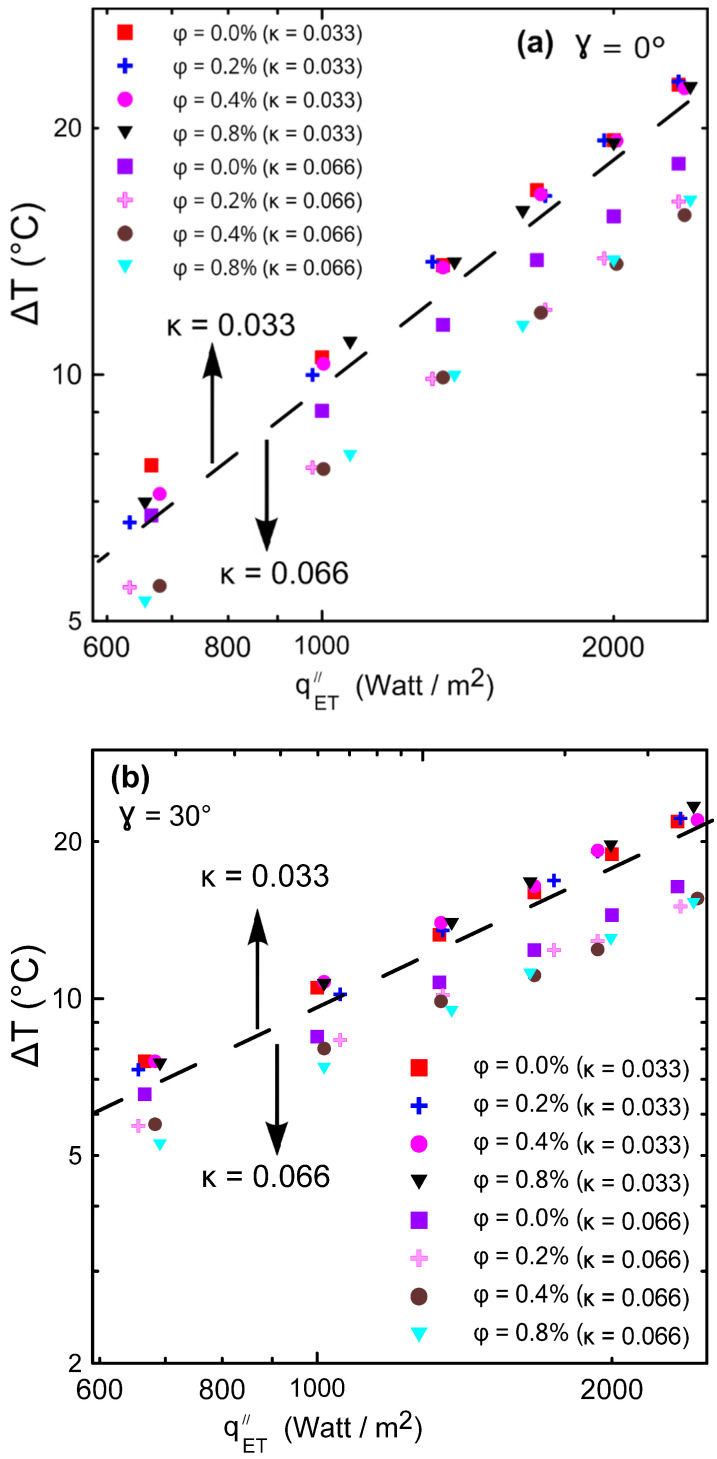
Temperature difference between cold and hot surfaces versus power input: (**a**) γ = 0°; (**b**) γ = 30°.

Figure 17a,b illustrate the temperature difference between cold and hot surfaces versus the input heat flux through the two used enclosures. It can be noticed that there were not many temperature difference (∆T) changes with increasing concentrations of the nanofluid (ϕ) for the small aspect ratio enclosure (#2, κ = 0.033) as shown in Figure 17a,b above the dashed line. On the other hand, for the large aspect ratio enclosure (#1, κ = 0.066), a large ∆T was observed at the same heat flux as the nanoparticles’ concentration changes as shown in Figure 17a,b below the dashed line. Figure 17a,b indeed explains why there was a large enhancement in Nu for the high aspect ratio enclosure compared to the small one as shown in Figure 15 and Figure 16, since the heat transfer coefficient was inversely proportional with ∆T. This may be attributed to the effect of strong natural convection in the high aspect ratio enclosure, which was almost absent in the small aspect ratio enclosures.

**Figure 18:**
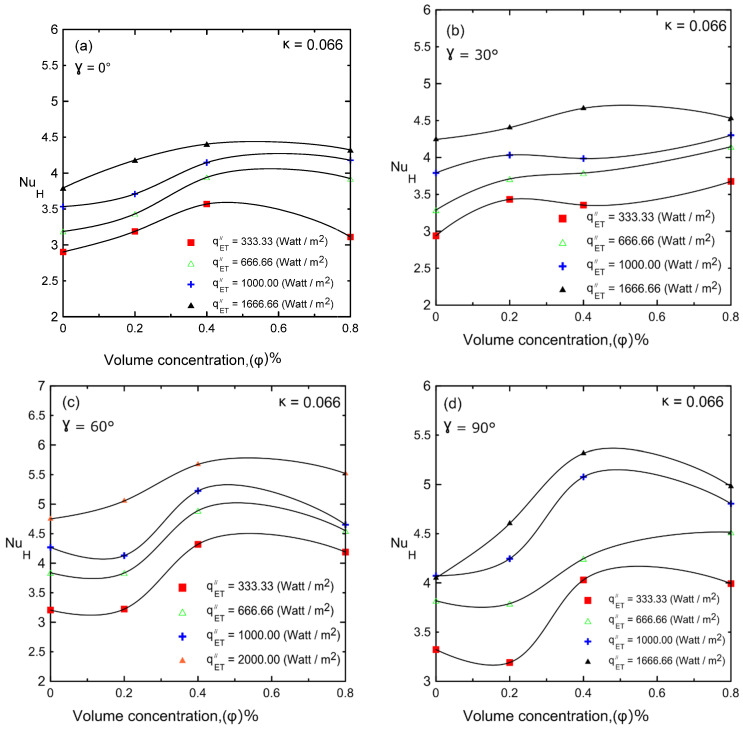
Nusselt number versus volume concentration of Al_2_O_3_–water nanofluid for different inclination angles: (**a**) 0°; (**b**) 30°; (**c**) 60°; (**d**) 90°.

The Nusselt number versus nanoparticle volume concentration is shown in Figure 18a–d for different heat fluxes at different inclination angles (i.e., 0°, 30°, 60°, and 90°) for enclosure number 1 (κ = 0.066). Natural convection heat transfer was significantly improved by adding nanoparticles with a volume concentration of up to 0.4–0.8%. As the nanoparticle concentration increased, the thermal conductivity and viscosity also increased and competed with each other. As a result, Nu decreased at high concentrations (μ increases). In many computational analyses, the heat transfer coefficient always increases as the concentration of nanofluid increases, although this phenomenon does not exist in the experimental works shown in the literature [4,9,10,30,35,46]. Therefore, the current experiments ensured that effect, which may be related to the fact that most of the computational analyses were 2D but the current experiments had a real 3D enclosure.

**Figure 19:**
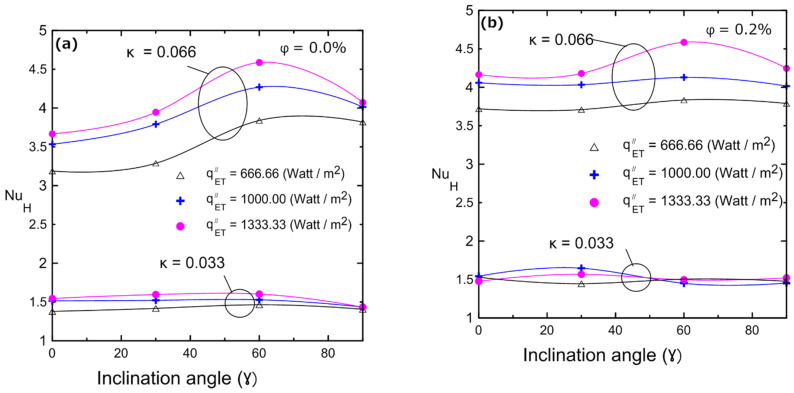

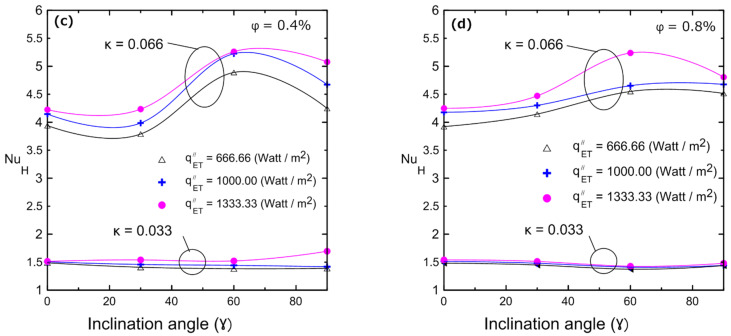
Nusselt number vs. inclination angle of the Al_2_O_3_–water nanofluid for different volume concentrations for both enclosure: (**a**) 0%; (**b**) 0.2%; (**c**) 0.4%; (**d**) 0.8%.

Variations in the Nusselt number with the inclination angle for different volume concentrations are illustrated in Figure 19: (**a**) 0%, (**b**) 0.2%, (**c**) 0.4%, and (**d**) 0.8% for two enclosures (κ = 0.033 and κ = 0.066). These figures indicate that in a large enclosure (κ = 0.066), the heat transfer coefficient and Nusselt number increased as the inclination angle increased, and they reached their maximum at 60° and then decreased again at 90°. This can be attributed to the developing buoyancy force and its effect on the velocity of the fluid and the developed vortices. This behavior was observed with all volume concentrations of the nanofluid in the large enclosure (κ = 0.066). Additionally, Figure 19 depicts the influence of inclination angle on the Nusselt number for enclosure number 2 (κ = 0.033). As a result of the low aspect ratio of the enclosure, the inclination angle had no effect on Nu, since the viscous forces overcame the weak buoyancy forces. The changes in natural convection heat transfer and Nusselt numbers can be physically interpreted as described numerically by many investigators [11,17,18,19], who showed streamlines and isothermal lines. Those flow patterns indicated that, for a horizontal cavity, the flow was dominated by two counter circulating cells (Rayleigh–Bénard cells) within the cavity. Indeed, the fluid moved in the middle of the cavity from the hot bottom surface towards the cold top surface and then fell away on the sides of the cavity, being pushed by the continually rising flow. As the tilt angle increased, the fluid ascended near the right side surface and fell near the left sidewall, creating a single anticlockwise direction circulating cell. It was shown that one vortex cell increased the induced velocity better than the two developed vortices in the case of the horizontal enclosure. This led to an increase in heat transfer coefficient and, hence, in Nu as indicated experimentally in Figure 19. This enhancement continued up to a maximum angle of 60° and then it reduced again at 90°, which could be attributed to a change in the flow field inside the enclosure to a boundary layer-type flow. Therefore, the experimental data shown in Figure 19 agree well with those of the numerical investigations [11,17,18,19]. It should be noted that the explanation for the counter rotating cells given above is valid for 2D enclosures. However, 2D physical analyses can still be considered for 3D enclosures as confirmed by Ravnik et al. [54], where the 2D approximation of the flow field was quite good and the 2D calculated Nusselt number values were quite close (within 8%) to the Nusselt number values obtained with a 3D simulation. Their study involved three-dimensional natural convection in an inclined enclosure using the boundary element method to study the free convection phenomenon in cubic and parallelepipedal enclosures.

**Figure 20:**
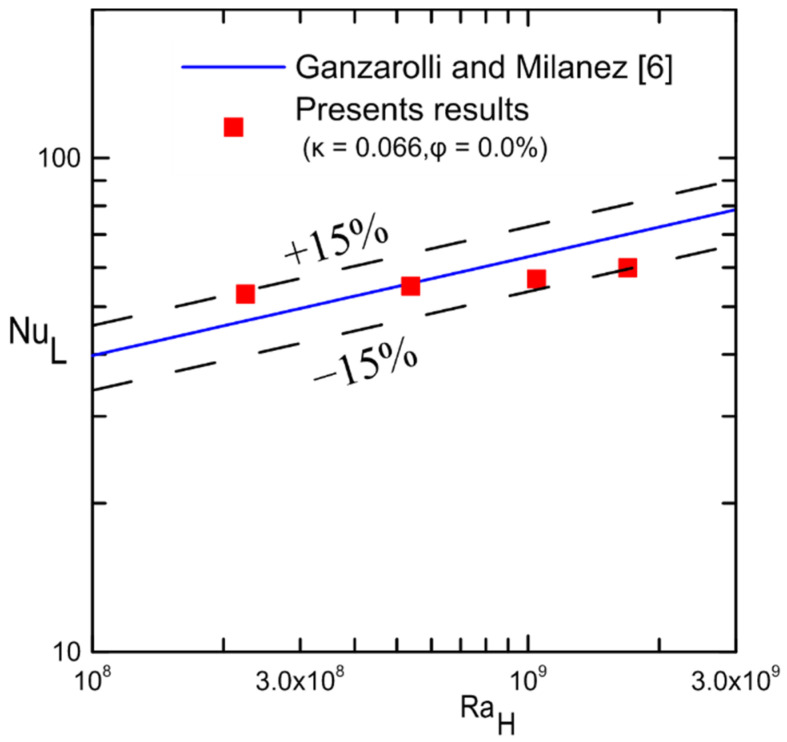
Comparison with the previous results in [6].

A comparison of the current experimental results for the cavity (κ = 0.066) filled with water and that of Ganzarolli and Milanez [6] is shown in Figure 20. This comparison shows that the experimental data were within ±15% of their theoretical results despite the difference in the boundary conditions. It should be noted that the Rayleigh number was used instead of ${\mathrm{Ra}}_{H}^{*}$, and the inside length of the cavity was used as a characteristic length in Nu calculation instead of the height of the cavity for the purpose of comparison.

## 4. Conclusions

A comprehensive experimental study was conducted on natural convection heat transfer inside enclosures filled with an alumina–water nanofluid with varying nanoparticle volume concentrations. Two square cuboid enclosures with aspect ratios of 0.033 and 0.066 were used at four different inclination angles: 0, 30, 60, and 90 degrees. Experimental measurements were performed to determine the nanofluid’s critical thermophysical properties. The thermal conductivity was measured at a variety of volume concentrations as well as the viscosity and density at temperatures ranging from 15 to 40 °C as a function of volume concentrations. The maximum differences of 6.61%, 16.36%, and 0.29% were obtained in thermal conductivity, dynamic viscosity, and density, respectively at a 1% volume concentration. The Nusselt number increased by increasing the nanofluid concentration over that of the base fluid up to 0.8% (Vol.) at the highest aspect ratio of the cavity enclosure number 1, κ = 0.066, while it did not change as much in the low aspect ratio enclosure number 1 (κ = 0.033), where the maximum average improvement in Nu was 27% at 60° and 0.4 Vol.%. Therefore, the aspect ratio can have a significant impact on heat transfer and convection performance. With an inclination angle between 0 and 90 degrees, it was shown that increasing tilt angle enhances the heat transfer coefficient at high aspect ratios (κ = 0.066), thereby enhancing the Nusselt number. Across all volume concentrations of nanoparticles, a tilt angle of 60° gave the highest Nusselt number. However, when the aspect ratio was small, as in κ = 0.033, the tilt angle almost had no effect on the Nusselt number, while pure conduction occurred with a Nu on the of order 1.

## Figures and Tables

**Table 1:** Specifications of the nanofluid as provided by the manufacturer for a concentration ratio 20% by mass.

Appearance	Translucent Liquid–White
Crystal structure and type	Gamma
PH value	>7
Original particle size	10 nm
Al_2_O_3_	20%
Solvent	80% water
Al_2_O_3_ Purity	99.99%

**Table 2:** Thermophysical properties of Al_2_O_3_ and water [37].

	Cp (J/Kg ∙ K)	ρ (kg/m^3^)	k (W/m ∙ K)	β × 10^− 4^ (K^−1^)
Water (base fluid)	4179	997	0.6	2.3
Al_2_O_3_ (nano particles)	765	3970	40	0.85

**Table 3:** Specifications of the distilled water device (HAMILTON WSC/4, twice distilled water machine, United Kingdom).

Output	4 Litres per Hour
Heaters	3 × 1.5 Kw Silica Heater/ 1 × 1.25 Kw Silica Heater
Power	220/240 v
Fuse	13 AMP
Minimum pressure supply	5 Psi
Ph	5.5–6.5
Electrical conductivity, µs/cm	<2.5
Resistivity, megohm-cm	0.66
Temperature	<35 °C
Thermostatic cut out	YES
Water supply cut out	NO
Wall or bench mountable	BOTH
Net weight	12 Kg

**Table 4:** Lab instruments used in this study.

Number	Equipment	Photo of the Instrument	Company	Manufacturer	Model
**1**	Thermal properties analyzer	* 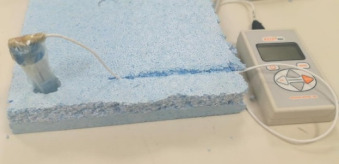 *	Decagon Devices	Pullman, WA, USA	KD2 pro
**2**	Kinematic viscometer	* 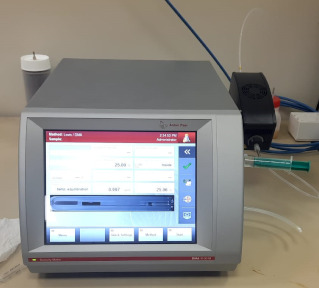 *	Anton Paar	Graz,Austria	SVM 2001
**3**	Ultrasonic prob	* 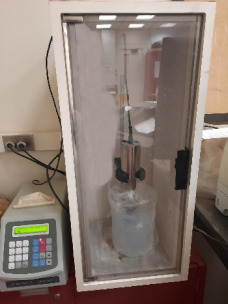 *	Qsonica	Newtown, CT,USA	Q-700
**4**	Electronic balance	* 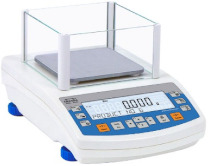 *	RADWAG	Radom,Poland	PS 600.R2
**5**	Magnetic Stirrer	* 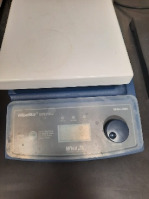 *	WiseStir	Wertheim, Germany	MSH-20D Set
**6**	Distilled water machine	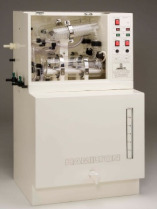	HAMILTON	Kent, United Kingdom	WSC/4D,Twice distilled water machine

**Table 5:** Enclosure parts.

Part Number	Part Description	Material	Dimensions (cm)
**1**	Enclosure cover (cold surface)	Copper	38×38×0.3
**2**	Gasket	Polyurethane	38×38×0.3
**3**	Two-way valve	Steel	Diameter = 0.635
**4**	Enclosure	Bakelite	$30\times 30\times \left(1\;\text{and}\;2\right)$
**5**	Gasket	Polyurethane	38×38×0.3
**6**	Enclosure cover (hot surface)	Copper	38×38×0.3
**7**	Electrical heater	Polyimide	30×30×0.0254
**8**	Insulation cover	Bakelite	38×38×3.6
**9**	Thermocouple	Type-K, self-adhesive	1.9×2.5×0.03
**10**	Insulation cover	Fiber glass	38×38×20

**Table 6:** The uncertainty of various parameters.

Quantity	Uncertainty (%)
$Q_{total}$	3.00
Q_Bkp_	8.07
Q_Bks_	12.11
Q_ET_	3.53
h_avg_	3.90
${\bar{Nu}}_{H}$	3.91
$Ra_{H}^{*}$	3.55

**Table 7:** Percent of deviation between the current experimentally measured thermal conductivity coefficient and Equation (13) of Maxwell [44] and Equation (14) of Williams [45] at different volume concentrations at ambient temperature.

Volume Concentrations (φ)%	Thermal Conductivity, Experimental Data (W/m ∙ °C)	Equation (13) of Maxwell [44](W/m ∙ °C)	Percent of Deviation	Equation (14) of Williams [45](W/m ∙ °C)	Percent of Deviation
0.10%	0.6245	0.6198	0.76%	0.6208	0.59%
0.20%	0.631	0.6215	1.52%	0.6236	1.18%
0.40%	0.642	0.6251	2.70%	0.6292	2.03%
0.50%	0.652	0.6269	4.00%	0.6321	3.15%
0.80%	0.661	0.6323	4.54%	0.6405	3.20%
1.00%	0.685	0.6426	6.61%	0.6529	4.91%

**Table 8:** Various viscosity models.

Author	Type of Model	Model Equation	
Einstein [47]	Theoretical	$\mu_{eff}=\left(1+2.5\varphi \right)\mu_{bf}$	(15)
Brinkman [48]	Theoretical	$\mu_{eff}=\mu_{bf}\frac{1}{{\left(1-\varphi \right)}^{2.5}}\;$	(16)
Ho et al. [49]	Experimental	$\mu_{eff}=\left(1+4.93\varphi +222.4\;\varphi^{2}\right)\mu_{bf}$	(17)
De Bruijn [50]	Theoretical	$\mu_{eff}=\left(1+2.5\varphi +4.698\;\varphi^{2}\right)\mu_{bf}\;$	(18)
Batchelor [51]	Theoretical	$\;\mu_{eff}=\left(1+2.5\varphi +6.5\;\varphi^{2}\right)\mu_{bf}$	(19)
Williams [45]	Experimental	$\mu_{eff}=\mu_{bf}\;exp\left(\frac{4.91\varphi }{0.2092-\varphi }\right)$	(20)

**Table 9:** Comparison of the experimentally measured dynamic viscosity and various viscosity models for Al_2_O_3_–water nanofluid.

Volume Concentrations (φ)%	Viscosity, Experimental Data (mPa ∙ s)	Einstein [47] (Equation (15)) (mPa ∙ s)	Percent of Deviation (% Change)	Brinkman [48] (Equation (16)) (mPa ∙ s)	Percent of Deviation (% Change)	De Bruijn [50] (Equation (18)) (mPa ∙ s)	Percent of Deviation (% Change)
0.10%	0.92871	0.93062	0.21%	0.93062	0.21%	0.93063	0.21%
0.20%	0.94319	0.93294	1.09%	0.93296	1.08%	0.93296	1.08%
0.40%	0.96740	0.93758	3.08%	0.93765	3.08%	0.93765	3.07%
0.50%	0.98572	0.93990	4.65%	0.94001	4.64%	0.94001	4.64%
0.80%	1.06496	0.94687	11.09%	0.94713	11.06%	0.94714	11.06%
1.00%	1.13760	0.95151	16.36%	0.95192	16.32%	0.95194	16.32%
**Volume Concentrations (φ)%**	**Viscosity, Experimental Data (mPa ∙ s)**	**Batchelor [51] (Equation (19))** **(mPa ∙ s)**	**Percent of Deviation (% Change)**	**Ho et al. [49] (Equation (17))** **(mPa ∙ s)**	**Percent of Deviation (% Change)**	**Williams [45] (Equation (20))** **(mPa ∙ s)**	**Percent of Deviation (% Change)**
0.10%	0.92871	0.93063	0.21%	0.93308	0.47%	0.95045	2.34%
0.20%	0.94319	0.93297	1.08%	0.93828	0.52%	0.97335	3.20%
0.40%	0.96740	0.93768	3.07%	0.94991	1.81%	1.02154	5.60%
0.50%	0.98572	0.94005	4.63%	0.95634	2.98%	1.04689	6.21%
0.80%	1.06496	0.94725	11.05%	0.97813	8.15%	1.12843	5.96%
1.00%	1.13760	0.95211	16.31%	0.99471	12.56%	1.18778	4.41%

**Table 10:** Comparison of the density values between the experiments and Equation (21) of Pak and Cho [53] for Al_2_O_3_–water nanofluid.

	Density of 1% (Vol.) Al_2_O_3_ (g/cm^3^)			Density of 0.8% (Vol.) Al_2_O_3_ (g/cm^3^)		
Temperature (°C)	Experiment	Equation (21) of Pak and Cho [53]	Percent of Deviation (%Change)	Experiment	Equation (21) of Pak and Cho [53]	Percent of Deviation (%Change)
15	1.026	1.0289	0.28%	1.0205	1.023	0.25%
20	1.0251	1.028	0.28%	1.0196	1.0221	0.24%
25	1.0239	1.0268	0.29%	1.0185	1.0209	0.24%
30	1.0225	1.0254	0.29%	1.017	1.0195	0.24%
35	1.0209	1.0238	0.28%	1.0154	1.0178	0.24%
40	1.019	1.022	0.29%	1.0135	1.016	0.24%
	**Density of 0.5% (Vol.) Al_2_O_3_ (g/cm^3^)**			**Density of 0.4% (Vol.) Al_2_O_3_ (g/cm^3^)**		
**Temperature (°C)**	**Experiment**	**Equation (21) of Pak and Cho [53]**	**Percent of Deviation (%Change)**	**Experiment**	**Equation (21) of Pak and Cho [53]**	**Percent of Deviation (%Change)**
15	1.0122	1.0141	0.18%	1.0098	1.0111	0.13%
20	1.0114	1.0132	0.17%	1.0089	1.0102	0.13%
25	1.0102	1.0120	0.17%	1.0078	1.0090	0.12%
30	1.0088	1.0106	0.18%	1.0063	1.0076	0.13%
35	1.0071	1.0089	0.18%	1.0047	1.0059	0.12%
40	1.0053	1.0071	0.18%	1.0028	1.0041	0.13%
	**Density of 0.2% (Vol.) Al_2_O_3_ (g/cm^3^)**			**Density of 0.1% (Vol.) Al_2_O_3_ (g/cm^3^)**		
**Temperature (°C)**	**Experiment**	**Equation (21) of Pak and Cho** [53]	**Percent of Deviation (%Change)**	**Experiment**	**Equation (21) of Pak and Cho** [53]	**Percent of Deviation (%Change)**
15	1.0044	1.0051	0.07%	1.0019	1.0022	0.03%
20	1.0035	1.0042	0.07%	1.0010	1.0013	0.03%
25	1.0023	1.0030	0.07%	0.9999	1.0001	0.02%
30	1.0009	1.0016	0.07%	0.9984	0.9987	0.03%
35	0.9993	1.0000	0.07%	0.9966	0.9970	0.04%
40	0.9975	0.9982	0.07%	0.9948	0.9952	0.04%

**Table 11:** The percentage of enhancement in Nusselt numbers at different tilt angles compared to pure fluid (Ф = 0%).

Angle	Minimum Enhancement in Nu for All Concentration	Average Enhancement in Nu for All Concentration	Maximum Enhancement for Nu in All Concentration	Concentration (Vol.%)	Average Enhancement for Nu% in Each Concentration
0°	9%	20%	32%	0.2%	17%
				0.4%	22%
				0.8%	23%
30°	5%	15%	32%	0.2%	10%
				0.4%	13%
				0.8%	24%
60°	3%	15%	35%	0.2%	3%
				0.4%	27%
				0.8%	24%
90°	3%	15%	30%	0.2%	3%
				0.4%	22%
				0.8%	20%

## Data Availability

The data presented in this study are available on request from the corresponding author. The data are not publicly available due to [it is part of a Ph.D. thesis results, after the thesis defense it will be available].

## Nomenclature

A	Enclosure surface area, m^2^
$A_{\mathrm{Bkp}}$	Surface area of the Bakelite plate above the heater, m^2^
$A_{\mathrm{Bks}}$	Surface area of the Bakelite sides, m^2^
H	Inside height of the cavity, m
h	Heat transfer coefficient, W m^−2^ k^−1^
I	Electrical current, ampere
K	Thermal conductivity, W m^−2^ k^−1^
Nu	Nusselt number, h H/k
*Q_total_*	Input power electrical, W
$Q_{Bkp}$	Heat lost by conduction through the lower Bakelite plate, W
$Q_{Bks}$	Heat lost by conduction through the Bakelite sides, W
$Q_{ET}$	Heat transfer by convection through the fluid, W
$q_{ET}^{\prime \prime }{}_{\;}$	Heat flux transfer by convection through the fluid, W/m^2^
R	Overall enclosure of the thermal resistances, K/W
R_Cppper_	Copper thermal resistances, K/W
$R_{\mathrm{fluid}}\;$	Fluid thermal resistances, K/W
${\mathrm{Ra}}_{H}^{*}{}^{\;}$	The modified Rayleigh number, $g\beta \;Q_{con}H^{4}\upsilon^{-1}\alpha^{-1}k^{-1}A^{-1}$
Ra →	Rayleigh number, $g\beta ∆{T\;H}^{3}\upsilon^{-1}\alpha^{-1}$
T	Temperature, °C
∆T	${\;T\;¯}_{avg\cdot h}-{\;T\;¯}_{avg\cdot c}$, °C
V	Voltage, volt
Greek symbols	
α	Thermal diffusivity, m^−2^ s^−1^
β	Coefficient for thermal expansion, K^−1^
δ	Bakelite thickness
ν	Kinematics viscosity, m^−2^ s^−1^
Δx	Thickness of copper, m
γ	Tilt angle of the cavity
κ	Aspect ratio
φ	Volume concentration
Subscripts	
avg·c	Cold surface
avg·h	Hot surface
bf	Base fluid
Bkp	Bakelite plate
Bks	Bakelite side
eff	Effective
exp	Experimental data
ET	Enclosure through
H	Characteristic length
nf	Nanofluid
p	Particle
Superscripts	
ـــ	Averaged quantity
